# Brain anatomy in Diplura (Hexapoda)

**DOI:** 10.1186/1742-9994-9-26

**Published:** 2012-10-11

**Authors:** Alexander Böhm, Nikolaus U Szucsich, Günther Pass

**Affiliations:** 1 Department of Evolutionary Biology, University of Vienna, Althanstrasse 14, 1090 Vienna, Austria

**Keywords:** Diplura, two-pronged bristletails, mushroom body, central body, 3D reconstruction, CNS, DC0, apterygote insects

## Abstract

**Background:**

In the past decade neuroanatomy has proved to be a valuable source of character systems that provide insights into arthropod relationships. Since the most detailed description of dipluran brain anatomy dates back to Hanström (1940) we re-investigated the brains of *Campodea augens* and *Catajapyx aquilonaris* with modern neuroanatomical techniques. The analyses are based on antibody staining and 3D reconstruction of the major neuropils and tracts from semi-thin section series.

**Results:**

Remarkable features of the investigated dipluran brains are a large central body, which is organized in nine columns and three layers, and well developed mushroom bodies with calyces receiving input from spheroidal olfactory glomeruli in the deutocerebrum. Antibody staining against a catalytic subunit of protein kinase A (DC0) was used to further characterize the mushroom bodies. The japygid *Catajapyx aquilonaris* possesses mushroom bodies which are connected across the midline, a unique condition within hexapods.

**Conclusions:**

Mushroom body and central body structure shows a high correspondence between japygids and campodeids. Some unique features indicate that neuroanatomy further supports the monophyly of Diplura. In a broader phylogenetic context, however, the polarization of brain characters becomes ambiguous. The mushroom bodies and the central body of Diplura in several aspects resemble those of Dicondylia, suggesting homology. In contrast, Archaeognatha completely lack mushroom bodies and exhibit a central body organization reminiscent of certain malacostracan crustaceans. Several hypotheses of brain evolution at the base of the hexapod tree are discussed.

## Background

Several recent neuroanatomical studies have covered aspects of the brain of Collembola [1], Archaeognatha [2] and Zygentoma [3-5]. The only major taxa of primarily wingless hexapods for which no recent information on their neuroanatomy is currently available are Protura and Diplura. Diplura, or two-pronged bristletails, are blind and wingless soil hexapods with long filiform antennae. Their cerci are pincer-like in the superfamily Japygoidea, long and filiform in Campodeoidea and short, bearing spinning glands, in Projapygoidea [6]. Molecular and morphological studies recover Diplura at almost all plausible tree nodes: As sister group to Ellipura (Entognatha hypothesis) [7], as sister group to Protura (Nonoculata hypothesis) [8-11], as sister group to Ectognatha (= Insecta s.s. = ‘true insects’) [12-14] and even outside of Hexapoda [14,15]. Moreover, monophyly of Diplura has been questioned on the basis of the structure of the reproductive system and ovaries (for reviews see [16,17]) and mitochondrial data [15,18]. The Projapygoidea are either placed in the suborder Rhabdura (together with Campodeoidea) [19] or are considered to be more closely related with Japygoidea [20]. The above collection of conflicting hypotheses underlines that Diplura could be one of the key taxa for understanding the early splits in the hexapod phylogenetic tree.

The use of neuroanatomical characters for phylogenetic reconstruction has flourished in the last decade (for a review see [21]). Characters concerning optic neuropils, the olfactory system and higher integration centers of the protocerebrum, such as the central complex and the mushroom bodies, have been used in many comparative analyses [4,5,22-30]. The brain neuropils of Diplura, namely of *Campodea* sp. and *Japyx* species, were first described in some detail by Holmgren [31]. His pupil Hanström added further observations for *Campodea* sp. and more detailed descriptions, including photomicrographs, of the brain of *Japyx purcelli* and *Japyx leae*[32,33]. Apart from later descriptions of neurosecretory cells and the associated corpora cardiaca and corpora allata [34-38], no further information is presently available on dipluran brain neuropils. To fill this gap we investigated the brain organization in representatives of *Campodea*, *Catajapyx* and *Metajapyx* using 3D reconstruction of semi-thin sections and antibody staining.

## Methods

### Animals

*Campodeaaugens* (Diplura: Campodeoidea) was collected in a deciduous forest (Vienna, N 48° 13.818’ E 16° 16.677’, WGS 84). *Catajapyx aquilonaris* (Diplura: Japygoidea), was collected on the southern slopes of the Leopoldsberg (Vienna, N 48° 16.542’ E 16° 20.756’, WGS 84). Animals were kept up to two months in small plastic boxes with a moist, soil covered plaster floor, either at room temperature or at 4°C. Occasionally tiny amounts of dry fish food or live Collembola were provided. For comparative investigations we used *Lithobius* sp. (Chilopoda: Lithobiidae) from the same sampling site as *Campodea augens*, *Metajapyx braueri* (Diplura: Japygoidea) collected at Leopoldsberg, as well as semi-thin sections of *Acerentomon maius* (Protura: Acerentomidae).

### Semi-thin sections and 3D reconstruction

Animals were anesthetized with carbon dioxide prior to dissection. Heads were cut off in PBS and subsequently transferred to Karnovsky’s fixative. Fixation lasted over night at 4°C, was ended by three washes in 0.1 M sodium cacodylate buffer and was finally followed by postfixation in 0.1 M OsO_4_. The specimens were then dehydrated in an ascending ethanol series and brought to epoxy resin (low viscosity resin, Agar Scientific Ltd.) via acetone. Ribbons of serial sections (1 *μ*m) were cut with a diamond knife (Diatome) on a Reichert Om U3 ultramicrotome [39]. The sections were stained between 30 s to 40 s at 65°C with diluted Richardson’s blue (1:9). Photos were taken on a Nikon Mikrophot FX-A microscope equipped with a Nikon DS-Fi1 digital camera (resolution: 1280 x 960 pixel). Two overlapping images of every section were captured and stitched together. Contrast enhancement (partially using the CLAHE plugin), stitching and alignment of images, manual segmentation and 3D reconstruction was done with TrakEM2 [40], a plugin for Fiji [41,42]. Final smoothing and rendering of the resulting 3D meshes was done with the open source 3D program Blender 2.49 [43].

All positional specifications are given in a coordinate system set up by the body axes, not the neuraxis. The figure orientation is indicated by small triangles containing the first letter of one of the following directional terms: anterior, posterior, dorsal, lateral.

### Antibodies

An affinity purified rabbit polyclonal anti-DC0 antibody [44,45] was generously supplied by D. Kalderon, Columbia University. This antibody is directed against the catalytic subunit of *Drosophila melanogaster* cAMP-dependent protein kinase A and was shown to preferentially label the mushroom bodies and Kenyon cell somata in representatives of various insect orders [3,26] and the hemiellipsoid bodies of the crustacean *Coenobita clypeatus*[46]. Specificity for other hexapods and arthropods is likely, given that the amino acid sequence of DC0 homologues is highly conserved across many animal phyla [47].

An unpurified rabbit polyclonal antibody against FMRFamide (Enzo life sciences) was used as a morphological marker. According to the manufacturer staining is abolished by pre-incubation with 10 nmol synthetic FMRFamide per ml diluted antibody. Since FMRFamide shares a protein motif with many other RFamides (e.g. [48]) it is likely that they are recognized by the used antibody as well. Thus we will refer to RFamide-like ir (immunoreactivity).

Antibodies raised in rabbit against *Diploptera punctata* allatostatin I (referred to as AS) and *Locusta migratoria* tachykinin II (referred to as TK) were kindly provided by H. Agricola, University of Jena. Both antibodies were previously characterized in [49] (AS) and [50,51] (TK). AS and TK were used to reveal potential layers of the central body as shown by [4,52].

As a control for unspecific binding of the secondary antibodies several specimens in each experiment were processed without adding primary antibodies, which resulted in no staining. Since no further specificity controls, for example Western blotting, were performed a chance remains that the used primary antibodies may also recognize closely related peptides in Diplura and we emphasize this by adding ‘-like’ after the primary antibody name when we talk about immunoreactivity.

### Immunolabeling

For antibody labeling heads were partially dissected and fixed with a 4% paraformaldehyde solution in 0.1 M phosphate buffered saline (PBS, pH 7.4) for 50 min up to 4 hours. Some specimens were fixed with 1% PFA in a 18.4 mM ZnCl_2_ solution and afterward washed in 10 mM HBS (HEPES-buffered saline) to avoid precipitation of ZnPO_4_[53]. After several washes in PBS, blocking was carried out for 1 h at room temperature in PBST (PBS with 0.3% Triton-X 100 added) containing 5% normal goat serum (Sigma-Aldrich) and 0.01% sodium azide. Primary antibodies (see above) were added to the blocking solution (anti-DC0, AS, TK: 1:250, anti-FMRFamide: 1:300). After, at most, 3 days incubation at 4°C and three washes in PBST, secondary antibodies (goat anti-rabbit conjugated to Alexa 568 (Molecular Probes) or Atto 633 (Sigma-Aldrich)) and phalloidin (labels F-actin; conjugated to Alexa 488; Molecular Probes) were diluted in fresh blocking solution and applied for another day. Nuclei were stained by adding DAPI (1:1000; Sigma-Aldrich). After washing in PBST specimens were dehydrated through an ethanol series and cleared in methylsalicylate. Five *Catajapyx aquilonaris* and over 30 *Campodea augens* were sucessfully processed. The whole mount preparations were examined with a Leica TCS SP 2 confocal microscope. Stacks were viewed and adjusted (brightness, contrast) with Fiji [41].

### Terminology

Neuroanatomical terminology is based on the proposals made by Richter et al. [54] whenever applicable.

## Results

### General brain anatomy

**Figure 1:**
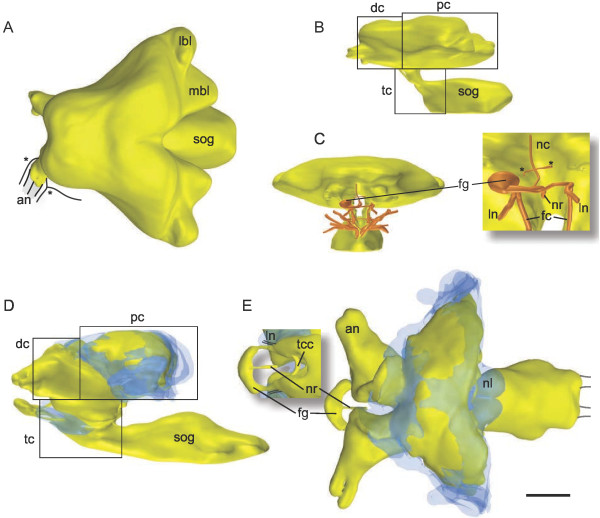
**Brain morphology of*Catajapyx aquilonaris* and*Campodea augens*.** A, B and C: Brain and sog of *Catajapyx aquilonaris*. **A)** Dorsal view (asterisks: motor nerves innervating intrinsic antennal muscles and muscles attached to the base of the antenna), **B)** Sagittal view. **C)** Anterior view including the nerves innervating the mouthparts. Inset: Frontal ganglion lying in the right half of the body. Asterisks: two branches of the nc. D and E: *Campodea augens*, cell somata blue, remaining tissue yellow. Somata covering the subesophageal ganglion not shown in the reconstruction. **D)** Lateral view. **E)** Dorsal view (inset: ventral view), connectives to the first thoracic ganglion indicated by black lines. Abbreviations: an antennal nerves, dc deutocerebrum, fg frontal ganglion, fc frontal connective, mbl median brain lobe, lbl lateral brain lobe, ln labral nerve, nc *nervus connectivus*, nr *nervus recurrens*, nl nuchal lobe, pc protocerebrum, sog subesophageal ganglion, tc tritocerebrum, tcc tritocerebral commissure. Scale bar: A, D, E: 100 *μ*m B, C: 70 *μ*m.

Diplura are characterized by a flattened, prognathous head capsule. The protocerebrum is tilted backward, lying immediately below the dorsal head cuticle. An anterodorsal position within the protocerebrum of a locust, for example, thus corresponds to a posterodorsal position in a dipluran brain. The sharp bend of the dipluran brain causes the deutocerebrum to be the most anterior part of the brain along the main body axis (Figure 1B, D). The tritocerebrum lies ventrally to protocerebrum and deutocerebrum on both sides of the esophagus or is fused with the subesophageal ganglion (see below). At our level of analysis no differences in external or internal brain morphology were found between *Catajapyx aquilonaris* and *Metajapyx braueri*. For a comparison between our terminology and the one used by Hanström [32] see Table 1. The terminology of an earlier account on the dipluran brain by Holmgren [31] is not included in this table but has been discussed by Hanström [32].

**Table 1:** Comparison between the terminology of Hanström and this report

**Hanström **[32]	**This report**
Stiel III [III]	mushroom body 1 [mb1]
Stiel IV + Glomeruli des	mushroom body 2 [mb2]
Stiels IV [IV + IV G]	
Stiel II [II]	mushroom body 3 [mb3]
Stiel I [I]	mushroom body 4 [mb4]
Zentrum Y [Y]	mushroom body 5 [mb5]
Globulizellen [Z]	Kenyon cells [kc]
Stielglomeruli [G, Dg, Vg]	calyces [clx1, clx2]
grosser Glomerulusballen des Deutocerebrums [Ab]	medio-ventral deutocerebral neuropil [mvdn]
Motorisches Antennalzentrum [M]	latero-ventral deutocerebral neuropil [lvdn]
Antennalglomeruli [Ag]	olfactory glomeruli [ogl]
Antennalkommissur	deutocerebral commissure [dcc]
Tractus olfactorio-globularis [Tr]	antenno-cerebral tract 1 [act1]
Medialkörper	median column
Nebenlappen [N]	lateral accessory lobes and/or anterio-lateral protocerebrum[lal, alp]

Only terms that are not used identically (for example Zentralkörper and central body) are listed. Our abbreviations and the abbreviations used in Hanström’s figures are given in square brackets.

**Figure 2:**
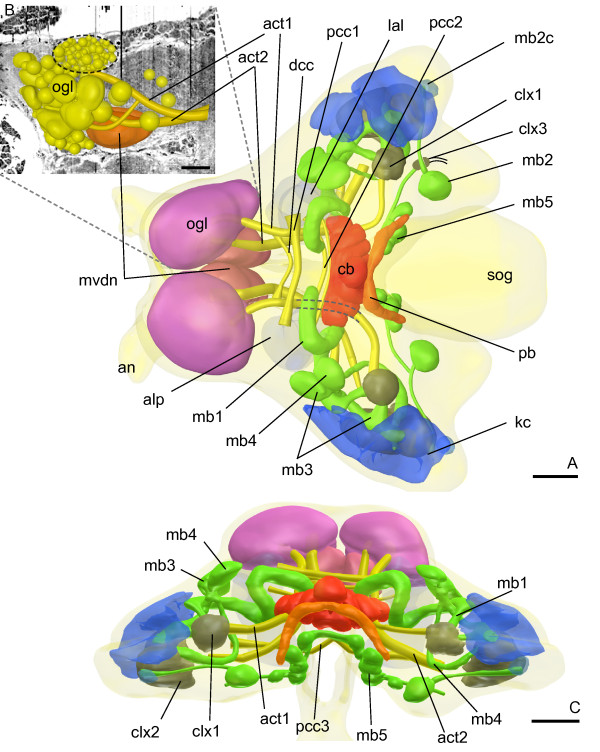
**Reconstruction of *Campodea augens* brain neuropils.** Dorsal view, cortex pale blue. Upper inset shows some of the calycal glomeruli as revealed by phalloidin (green) and nuclear (red) staining. Lower inset shows the protruding median column from anterior. Abbreviations: alp antero-lateral protocerebrum, an antennal nerves, act[x] antenno-cerebral tract x, cb central body, kc Kenyon cells, fg frontal ganglion, lal lateral accessory lobe, mb[x] mushroom body number x, mb1’ branch of mb1 connected to clx, ogl olfactory glomeruli, pb protocerebral bridge, pi *pars intercerebralis*, pcc[x] protocerebral commissure x, clx calyx, sog subesophageal ganglion. Scale bars: reconstruction: 50 *μ*m, upper inset: 20 *μ*m.

**Figure 3:**
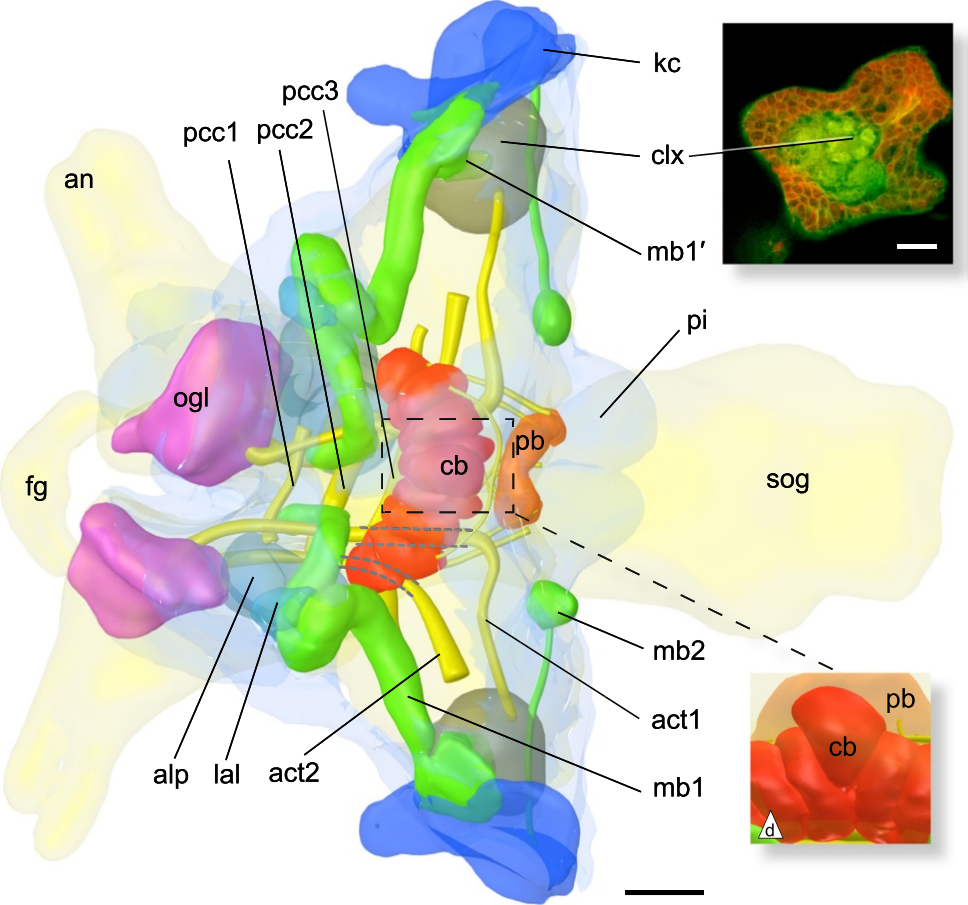
**Neuropil organization of the brain in *Catajapyx aquilonaris* and *Metajapyx braueri*. A)** Overview of the brain of *Catajapyx aquilonaris*. Mushroom bodies indicated by asterisks. **B** and **C)** Cross sections of the deutocerebrum of *Metajapyx braueri*. Group of small lateral olfactroy glomeruli indicated by arrow. One of the larger dorsal olfactory glomeruli is marked by a plus sign. The medio-ventral (mvdn) and latero-ventral (lvdn) glomerular deutocerebral neuropils are indicated by one or two asterisks, respectively. **D)** Cross section through the central body of *Metajapyx braueri*. The median column (asterisk) protrudes above the others. **E)** Deutocerebrum of *Catajapyx aquilonaris*. Detail of neurites (arrow) entering the mvdn, a cup-shaped medial neuropil (dashed line). This structure is separated from its counterpart in the opposing hemisphere by a connective tissue layer. **F)** Protocerebral bridge of *Metajapyx braueri*. Abbreviations: alp antero-lateral protocerebrum, amp antero-median protocerebrum, act1 antenno-cerebral tract 1, cb central body, kc Kenyon cells, mvdn medio-ventral deutocerebral neuropil, lvdn latero-ventral deutocerebral neuropil, pb protocerebral bridge, clx[x] calyx x. Scale bars: A: 50 *μ*m, B, C: 25 *μ*m, D, E, F: 20 *μ*m.

In dorsal view the overall shape of the dipluran brain is triangular with the highest width in the posterior protocerebrum (Figure 1A, C, E). The most conspicuous shape differences pertain to the hind margin of the protocerebrum and the spatial relation of the brain to extrinsic antennal muscles. The hind margin of the brain of *Catajapyx aquilonaris* is dominated on each side by two lobes with a large groove between the median lobes (Figure 1A). In contrast, in *Campodea augens* postero-medial cell bodies of the *pars intercerebralis* form two adjacent paraboloidal lobes called ‘Nackenloben’ (nuchal lobes) by Holmgren [31] (Figures 1E and 2). Additionally the postero-lateral protocerebrum of *Campodea augens* forms a pronounced groove where head muscles, extending to the base of, and into, the antennae, lie. In all species relatively large areas of the dorsal surface of the protocerebrum and parts of the deutocerebrum are not covered by a cortex of cell somata (Figures 1E and 3B).

The antennal nerves vary in number among *Campodea augens* and the investigated japygids. In *Campodea augens* two antennal nerves of equal diameter are present on each side, whereas in japygids two additional small motor nerves occur [32] (Figure 1A). A branch of the lateral motor nerve does not enter the antenna but extends posteriorly into the head and innervates two muscles attached to the base of the antenna (Figure 1A).

Frontal connectives, originating from the tritocerebrum, enter the frontal ganglion. Large differences among species are present regarding the frontal ganglion and its connections to the brain. In *Catajapyx aquilonaris* the spheroidal frontal ganglion is located asymmetrically in the right body half (Figure 1C). It is connected with a *nervus recurrens* and its frontal connectives, which descend in parallel to the circumesophageal connectives (Figure 1C). The frontal connectives originate at an apical portion of the subesophageal ganglion, in close vicinity to the origin of the nerves supplying the mouthparts. The labral nerves branch from the frontal connectives at the level of the frontal ganglion (Figure 1C, inset). No free tritocerebral commissures are present. The origin of the frontal ganglion connectives and the absence of a discernible tritocerebral region in the brain suggests that the tritocerebrum is, at least to a great extent, fused with the subesophageal ganglion. In addition to the frontal connectives, the frontal ganglion is connected to the brain by a small, unpaired *nervus connectivus*[32]. This nerve gives off two small branches (Figure 1C) that probably supply nearby cibarium dilators. Further dorsally this *nervus connectivus* is joined by two tegumentary branches [31] and enters the deutocerebrum. Upon this entry the nerve could not be traced any further.

In contrast, the tritocerebrum of *Campodea augens* is situated on both sides of the esophagus and exhibits one free tritocerebral commissure (Figure 1E, inset). Apart from a *nervus recurrens* and the frontal connectives, no other nerves could be observed that connect to the frontal ganglion, which is symmetrical and crescent shaped (Figure 1E). The labral nerves are found next to the frontal connectives (Figure 1E, inset).

### Protocerebrum

**Figure 4:**
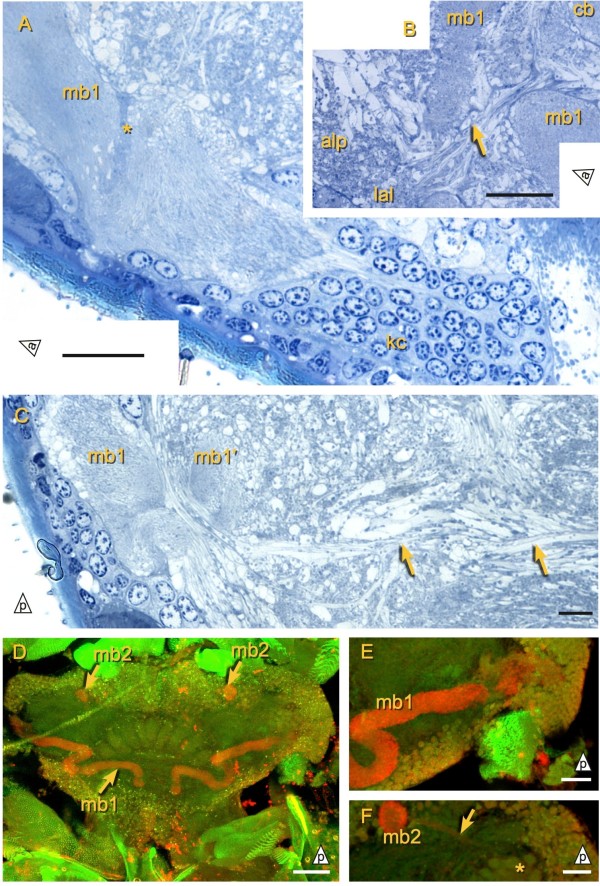
**Reconstruction of *Catajapyx aquilonaris* and *Metajapyx braueri* brain neuropils. A)** Dorsal view, *Catajapyx aquilonaris*. **B)** Reconstruction of the deutocerebrum of *Metajapyx braueri*. Dashed line: lateral group of smaller olfactory glomeruli. **C)** Posterior view, *Catajapyx aquilonaris*. Abbreviations: alp antero-lateral protocerebrum, an antennal nerves, act[x] antenno-cerebral tract x, cb central body, dcc deutocerebral commissure, kc Kenyon cells, lal lateral accessory lobe, mb[x] mushroom body number x, mb2c large cells at the base of the peduncle of mb2, mvdn medio-ventral deutocerebral neuropil, ogl olfactory glomeruli, pb protocerebral bridge, pcc[x] protocerebral commissure x, clx[x] calyx x, sog subesophageal ganglion. Scale bars: A, C: 50 *μ*m B: 30 *μ*m.

In all investigated species three larger protocerebral commissures (pcc) stand out among several smaller ones: two (pcc1, pcc2) connect the right and left parts of the alp (antero-lateral protocerebrum) and extend immediately above act1 and act2 (antenno-cerebral tracts), respectively (Figures 2 and 4). Another large commissure (pcc3) is located under the central body and connects both hemispheres of the lateral protocerebrum.

**Figure 5:**
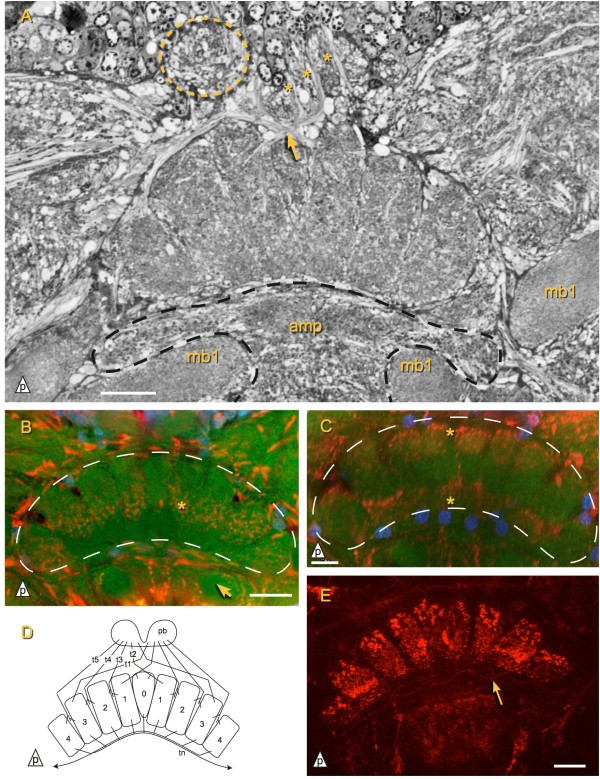
**Mushroom bodies of *Campodea augens*. A)** Proximal part of mushroom body 1 (mb1) with intrinsic Kenyon cells (kc). A darkly stained region (asterisk) partially separates the peduncle of mb1 from the neuropil at its base. **B)** Connection (arrow) of the central body with the lateral accessory lobes (lal), which are in turn connected with the antero-lateral protocerebrum (alp). **C)** Possible connection of axons passing below mb1 and mb1’ (left arrow; mb1’: branch of mb1 extending to the calyx) and axons of the act2 (antenno-cerebral tract 2, right arrow). **D)** DC0-like ir (red) and phalloidin staining (green) reveal mb1 and the globular lobe of mb2. E) Detail of mb1. **F)** Detail of mb2 and its thin peduncle (arrow) which originates from lateral Kenyon cells and closely passes by the calycal glomeruli (asterisk). Scale bars: A, B, E, F: 20 *μ*m, C: 10 *μ*m, D: 50 *μ*m.

Small protocerebral lobes (lal, lateral accessory lobes) are connected to the central body (Figure 5B), and probably to each other, by a small neurite bundle directly in front of the central body. Anterior of each lal lies the antero-lateral protocerebrum (alp) of the respective hemisphere (Figures 2, 4 and 5B). The alp is intimately connected with the lal, as well as with the remaining protocerebrum and the tritocerebrum.

### Central complex

**Figure 6:**
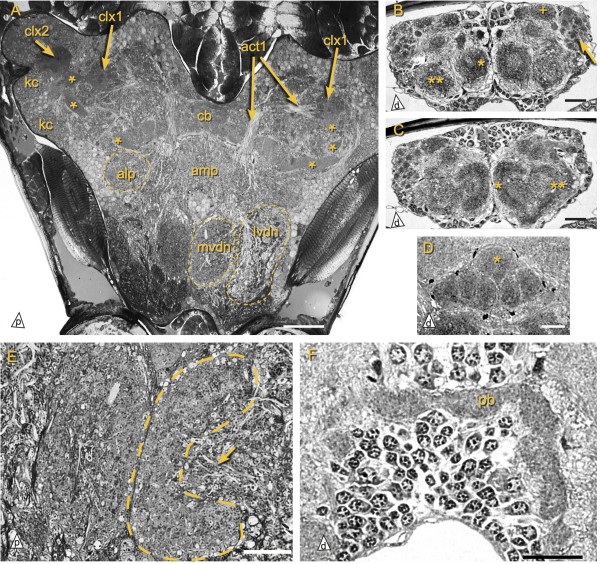
**Central complex of *Campodea augens*. A)** Horizontal section showing the protocerebral bridge (pb, on one side indicated by dashed line) and the central body (cb). Tracts (asterisk) extending through the pb form a chiasma (arrow) before entering the cb columns. mb1: mushroom body 1. amp: anterio-median protocerebral neuropil (dashed line). **B)** TK-like ir is observed in a middle layer (asterisk) of the central body. Arrow: mb1 Red: TK, green: phalloidin, blue: DAPI. **C)** AS-like ir present predominantly in a posterior and an anterior layer of the central body (asterisks). Red: AS, green: phalloidin, blue: DAPI. **D)** Tentative diagram of the central body connectivity. Tracts (t[x]) passing through the protocerebral bridge supply the cenctral body median column (0) and lateral columns (1 to 4). tn: tangential neurons **E)** RFamide-like ir in the central body. Note neurites innervating the columns from their anterior face (arrow). Scale bars: D: 25 *μ*m, others: 20 *μ*m.

The crescent-shaped central body is made up of 4 lateral columns on each side (Figure 6) and an unpaired median column that protrudes spherically above the others (Figures 2 and 3D). In addition to this latero-medial compartmentalization, the dipluran central body is also differentiated along the antero-posterior axis. A division into distinct layers is pronounced in the semi-thin sections but in *Catajapyx aquilonaris* the lateral column’s neuropil is less dense anteriorly and in *Campodea augens* a middle layer exhibits a higher density of darkly stained granules.

Antibody staining against AS, TK, and FMRFamide in *Campodea augens* revealed three central body layers along the antero-posterior axis: The middle layer is TK-like ir (Figure 6B), whereas AS-like ir is strongest in the anterior and posterior layer (Figure 6C). All central body columns exhibit RFamide-like ir which is not clearly layered but still stronger in the posterior layer and between the middle and anterior layer (Figure 6E). Furthermore anti-FMRFamide immunostaining reveals a class of tangential neurons that invade the central body from the anterior side and have arborizations in all columns (Figure 6D, E).

The protocerebral bridge lies posterior of the central body and is more slender in the investigated Japygidae (Figure 3F) than in *Campodea augens* (Figure 2). Tracts pass through the protocerebral bridge and arborize in the central body (Figure 6A, D). In principle these tracts correspond to the w, x, y and z tracts of pterygotes [55], but since at least five tracts per hemisphere are present in *Campodea augens* this correspondence is not one-to-one and we name the tracts t1 to t5. The medial four neurite bundles (t1 and t2, Figure 6D) form a chiasma while the others are homolateral. Since other protocerebral tracts also extend along the posterior border of the central body, the target of the innermost bundles could not be unambiguously identified in the semi-thin sections, but is likely found in column three (counting from median, the median column being column zero) and/or its neighboring columns (Figure 6D). Columns zero (the median column) and one are targeted by the second set of heterolateral tracts (t2) while the remaining homolateral tracts (t3 to t5) seem to enter the central body along the border between two respective adjoining columns, likely innervating both of them.

### Mushroom bodies

**Figure 7:**
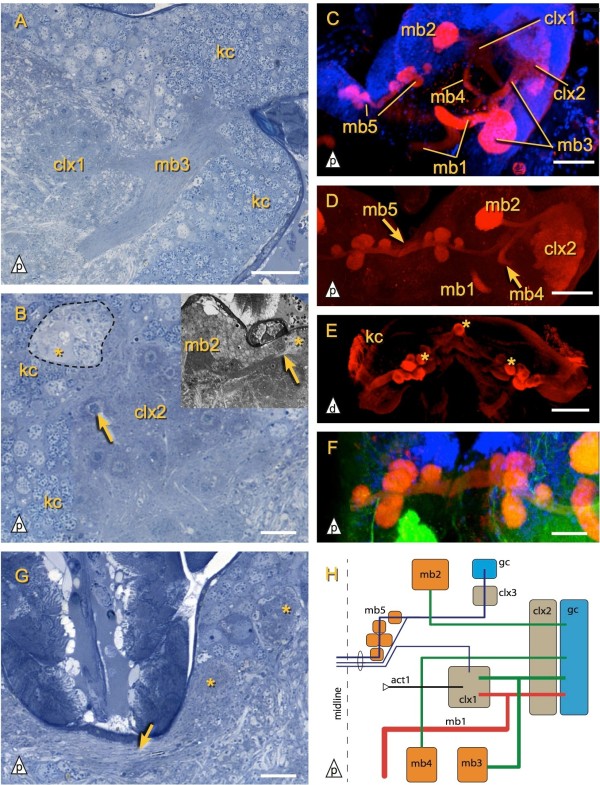
**Mushroom bodies of *Catajapyx aquilonaris*. A)** mb3 with Kenyon cells and connection to calyx 1. **B)** Calyx 2 showing cores of small darkly stained granules (arrow). Group of cells (dashed line, asterisk marks large single cell) with weakly stained cytoplasm. Inset: neurites of the same cell group (asterisk) extending through the middle of the peduncle of mb2 (arrow). **C, D, F)** Brain of *Catajapyx aquilonaris* stained with an antibody against DC0 (red), DAPI (blue) and phalloidin (green). Note that mb5 is connected across the midline. **E)** For comparison: DC0-like ir in *Lithobius* sp. is strong in the Kenyon cells and Trauben of the mb (asterisk). **G)** Midline spanning commissure-like connection (arrow) of mb5 and two of its Trauben (asterisk). **H)** Diagram of the mushroom body system in *Catajapyx aquilonaris*. Crossing lines of different color are not connected. Abbreviations: act1 antenno-cerebral tract 1, kc Kenyon cells, mb[x] mushroom body number x, clx[x] calyx x. Scale bars: A, F: 20 *μ*m, B, G: 10 *μ*m, C, D: 40 *μ*m, E: 100 *μ*m.

The mushroom bodies (mb) of the investigated diplurans were identified based on their morphology and on their DC0-like ir. We term sets of a peduncle and attached lobes mb1 to mb5. The calyces are named separately (clx1, clx2) because they are shared by more than one peduncle (Figure 7H). With our methods it was not possible to determine the arborization pattern of the Kenyon cell dendrites in the calyces. The mushroom bodies of the investigated Diplura can be grouped into three categories: (i) long peduncles of constant thickness which form a characteristic loop (mb1 Figures 2, 4 and 5) (ii) peduncles with globular terminal lobes (mb2 Figures 3 and 4D; mb3, mb4 Figures 4 and 7A, C, D) (iii) five interconnected spherical lobes (‘Trauben’) in each half of the brain, with a connection across the midline (mb5 Figures 4 and 7C, D, F, G). In *Campodea augens* only mb1 and mb2 are present, whereas examined Japygidae have the full set of five mushroom bodies (Figure 7H).

#### Catajapyx aquilonaris

The peduncle of mb1 originates from a group of small diameter Kenyon cells in the postero-lateral region of the protocerebrum. This anterior Kenyon cell group is separated from posterior ones by a band of non-Kenyon cell somata. Shortly after leaving the cell cortex one branch of mb1’s peduncle is connected to the spherical calyx 1 (clx1, Hanströms ‘Stielglomeruli’), which contains glomeruli with an outer DC0-like ir ring and an inner non DC0-like ir core (average diameter of glomeruli 6.3 *μ*m, Figure 3A, 4 and 7A). clx1 is the target of act1 (Figure 3A). The main peduncle extends toward the central body, where it makes a loop, first turning upward, and then downward again, before ending bluntly in front of the central body. At the base of the peduncle of mb1, mb2, mb3, and mb4 lie groups of cells with a less distinctly stained cytoplasm in semi-thin sections (only shown for mb2, asterisk Figure 7B). These groups contain one large cell (diameter up to 16 *μ*m; average diameter of Kenyon cells 3.2 *μ*m) and a varying amount of smaller cells. The core of the peduncle of mb2 consists of neurites from this cell group (Figure 7B, inset). The thin peduncle of mb2 has a globular terminal lobe at the posterior margin of the brain (Figure 7B, C, D).

Both mb3 and mb4 originate in the lateral protocerebrum and terminate with lobes immediately adjacent to the dorsal neurilemma (Figure 4). The peduncles of mb3 and mb4 pass through calyx 2 (clx2), which is consisting of glomeruli lying just medial of the Kenyon cell layer. These glomeruli are ventrally indistinguishable but dorsally a posterior group and an anterior group can be discerned. As in clx1, the glomeruli of clx2 have a dense core (Figure 7B) that is not DC0-like ir. While the neuropil of the lobe of mb3 is very dense and strongly stained by Richardson’s stain, mb4 has a less dense lobe that also exhibits less DC0-like ir. The peduncle of mb3 is, like mb1, connected to clx1 while the peduncle of mb4 describes a curve around clx1 without being connected to it (Figures 4 and 7C, D).

The mb5 (‘Zentrum Y’ of Hanström [32], ‘Ocellar-glomeruli’ and ‘unterer Glomerulus’ of Holmgren [31]) consists of five ‘Trauben’ (German for grapes, introduced for *Lepisma saccharina* mushroom bodies by [56]) on each side and is located below the protocerebral bridge (Figures 4 and 7C, D, F, G). These ‘Trauben’ are connected across the midline and are supplied by a thin peduncle, coming from a small group of Kenyon cells in the posterio-lateral protocerebrum and passing through calyx 3 (clx3, Figure 4A), which consists of very small glomeruli. A small tract connects mb5 to clx1 (Figure 7H).

All mb’s are DC0-like ir (Figure 7C, D, F). The strongest staining was observed in the lobes of mb2 and mb3. To a lesser extent the calyces clx1, clx2 and clx3 are DC0-like ir as well, whereas the staining of Kenyon cell somata comparatively weak.

#### Campodea augens

Kenyon cells in the postero-lateral protocerebrum give rise to two mushroom bodies, mb1 and mb2 (Figure 2 and 5D). Their shape, position and DC0-like ir (Figure 5D, E, F) are very similar to their counterparts in *Catajapyx aquilonaris*. Only one calyx (clx) is present. Like clx1 in *Catajapyx aquilonaris* it is connected with both act1 and mb1. In mb1 a core of longitudinally arranged neurites is surrounded by orthogonal ones (TEM data, not shown). Similar cores have also been observed in *Thermobia domestica* and in several pterygote insects [3,26]. Kenyon cell soma morphology is uniform and larger cells at the base of the peduncles, like in *Catajapyx aquilonaris*, were not found.

### Deutocerebrum

Two groups of olfactory glomeruli are present in the deutocerebrum of both *Catajapyx aquilonaris* and *Metajapyx braueri*. One of them consists of approximately one hundred relatively small glomeruli (average diameter 7 *μ*m) in lateral position (Figure 4B, dashed line). Medial of this aggregation, olfactory glomeruli of various sizes, up to 30 *μ*m diameter, are found (Figure 3B and 4B). Apart from the olfactory glomeruli two large cup-shaped neuropils are present: a medio-ventral and a latero-ventral deutocerebral neuropil (mvdn, lvdn; Figure 3A, B, C). The above mentioned small antennal motor nerves are connected with the lvdn [32]. While the mvdn has a dense neuropil layer (Figure 3E), the lvdn is generally less uniform and of lower neuropil density. In certain cross sections of *Metajapyx braueri* the lvdn is almost symmetrical to the mvdn and they appear to be connected (Figure 3C). Both lvdn and mvdn receive antennal afferents and are connected with the tritocerebrum by large profiles.

**Figure 8:**
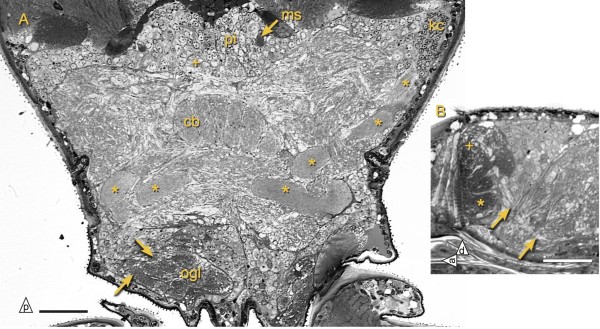
**Brain neuropils of *Campodea augens*. A)** Protocerebral bridge (plus sign), parts of mushroom body mb1 (asterisk). Arrows indicate two of the olfactory glomeruli. **B)** Parasagittal section showing large and irregularly shaped olfactory glomeruli (asterisk) and a less dense region with smaller olfactory glomeruli (plus sign). Arrows indicate third antenno-cerebral tract. Abbreviations: ogl olfactory glomeruli, cb central body, kc Kenyon cells, ms muscle, pi *pars intercerebralis*. Scale bars: 50 *μ*m.

The studied species of Japygidae differ from *Campodea augens* in both number and shape of observed deutocerebral neuropils. In *Campodea augens* two different types of olfactory glomeruli exist: four to five large, elongated, ventral ones and small spheroidal glomeruli dorsally (Figure 8). Neuropils equivalent to the mvdn and lvdn of japygids could not be found in *Campodea augens*.

A small commissure (dcc) connects both hemispheres of the deutocerebrum and several pairs of tracts connect the olfactory glomeruli with the protocerebrum: act1 passes directly under the central body before making a sharp turn toward the lateral protocerebrum where it ends at the clx1. A second tract, act2, extends to the lateral protocerebrum as well. Some of its axons appear to pass under the basal region of mb1 from which the small peduncle branches off and extends to the calyx (Figure 5C). Only in *Campodea augens* could another tract, act3 (Figure 8B; not included in the reconstruction), be traced from the olfactory glomeruli, along the ventral border of the protocerebrum, to the postero-lateral protocerebrum where it runs along axons of act2.

## Discussion

### Brain evolution within Diplura

Many brain structures shared exclusively by Campodeidae and Japygidae provide strong evidence for the monophyly of Diplura. The most striking of these are (i) the division of the central body into nine columns, with the median column protruding above the others, (ii) a mushroom body peduncle with a conspicuous loop, ending in front of the central body (mb1), (iii) the presence of a narrow peduncle with a globular lobe at the posterior end of the protocerebrum (mb2). Although looped lobes and globular lobes have been described in other species (e.g. Figure EightD in [57]), the spatial relationship of mb1 and mb2 to other neuropils, together with their shape, is specific for Diplura.

Of all studied neuroanatomical structures the deutocerebral neuropils and the mushroom bodies seem to have the highest information content for internal dipluran relationships, since they differ between Campodeidae and Japygidae in both organization and complexity.

The deutocerebrum of Diplura contains two groups of olfactory glomeruli: one consists of small spheroidal glomeruli while the other contains large elongated glomeruli in *Campodea augens* and numerous large, mostly spheroidal glomeruli in the investigated japygids. With the methods employed by us we cannot rule out that some of the putative olfactory glomeruli may be involved in processing thermoreceptive or hygroreceptive afferents, as, for example, has been suggested for a glomerular ventral neuropil in the deutocerebrum of Archaeognatha [2]. The deutocerebrum of Japygidae additionally contains two large ventral neuropils (mvdn and lvdn). Given the connection of the lvdn with antennal motor nerves and with the mvdn, these neuropils likely fulfill mechanosensory and motor functions, as does the antennal mechanosensory and motor center in insects [58]. In *Campodea augens* these functions are likely performed by a more diffuse deutocerebral neuropil that was not identified by us.

The higher complexity of the deutocerebrum of Japygidae is also reflected in the mushroom bodies. It can be speculated that this high anatomical complexity leads to enhanced olfactory, learning and memory capabilities advantageous for the predatory lifestyle of japygids [6].

While molecular data supports a closer relation of Projapygoidea with Japygoidea [20], morphological data presently cannot resolve the position of Projapygoidea since they exhibit a ‘balanced mix’ of characters of Campodeoidea and Japygoidea [6]. Preliminary data (AB, unpublished) on the brain anatomy of *Octostigma sinensis* suggests that Projapygoidea largely correspond with Japygidae in organization of the mushroom bodies and of the deutocerebrum. Currently no unambiguous polarization is possible, but outgroup comparison at present stands favors the high number of japygid mushroom bodies to be apomorphic.

### Homologization and polarization of brain characters

A conspicuous feature of the general brain anatomy in all studied Diplura is the backward tilted protocerebrum below the dorsal head cuticle. A comparable condition is likewise present in Protura, where the protocerebrum extends even into the thorax [59,60]. While tempting at first glance, this similarity should not be assessed as a synapomorphy supporting a sister group relationship of Diplura and Protura (Nonoculata hypothesis). Similar arrangements occur in many groups, for example Remipedia [22], Chilopoda [61] or some Collembola [1], and likely evolved convergently in response to spatial constraints imposed by the head capsule or internal components, such as muscles.

#### Mushroom bodies

The term ‘mushroom body’ is used for brain neuropils of annelids, onychophorans, and various arthropods [62-64]. Recent studies suggest that the hemiellipsoid bodies of malacostracan crustaceans are homologous to the calyces of the insect mb’s [65] or that the underlying neuronal circuits are homologous between insects and malacostracans [46,66]. The Cephalocarida, closely affiliated with Remipedia according to [67], have large mb’s [65,68] (also termed ‘multi-lobed’ complex by [22,65]) that form ‘Trauben’ like the mb’s of *Lithobius* sp. (Figure 7E), Japygidae, and Zygentoma [3]. Branchiopoda, which some recent molecular studies (e.g. [8,11]) found as a sistergroup to Hexapoda, have likely lost hemiellipsoid bodies along with other brain centers typical for Malacostraca and Hexapoda [30].

In neopteran insects [69], as well as Zygentoma [3,57,70] and the malacostracan *Coenobita clypeatus*[66], calyces contain so-called microglomeruli. The calycal glomeruli of Diplura, like microglomeruli, consist of a core enclosed by a dense shell of Kenyon cell neurites (especially well visible in *Catajapyx aquilonaris*). We hypothesize that the inner core contains presynaptic boutons of projection neurons extending from the olfactory glomeruli through the act’s to the calyces, as in Neoptera [69].

The observed large cells at the base of the peduncles of *Catajapyx aquilonaris* could be mushroom body neuroblasts that generate new Kenyon cells. The core of mb2 in *Catajapyx aquilonaris* (Figure 7B) could consist of neurites of such newborn Kenyon cells. While we did not demonstrate that these cells are mitotically active, proliferative cells giving rise to intrinsic mushroom body neurons occur in several insects after embryogenesis and into adulthood (Zygentoma: [3], Orthoptera: [71], Lepidoptera: [72,73]).

A comparison with data on other primarily wingless hexapods allows for no clear polarization of characters of the mushroom bodies. Although mushroom bodies were reported as present in Protura [59], this finding awaits independent confirmation. In Collembola, Kollmann et al. [1] found evidence for simple mushroom bodies in one out of three investigated species. Mushroom bodies are absent in Archaeognatha, but are present in Zygentoma and most pterygotes [26]. The system of mushroom bodies in Japygidae is among the most complex of all hexapods: Three mushroom bodies (mb3, mb4 and mb5) are present in addition to mb1 and mb2, which also occur in *Campodea augens*. This unexpected variability of mushroom body structure and complexity among primarily wingless hexapods, ranging from extremely complex to completely absent, leaves character polarization in many cases hardly possible.

Only in some features does the observed distribution of character states allow for preliminary character polarization. The mb5 of Japygidae is the only mushroom body known in hexapods that has a commissure-like connection across the midline. Since mushroom bodies connected across the midline likewise occur in some chilopods (Figure 7E; [74]), cephalocarids [65,68], onychophorans and chelicerates [29] this most probably represents either the plesiomorphic condition or a reversal to the ancestral state. The unpaired mushroom body midline neuropil of *Lithobius* sp. (Figure 7E) and Cephalocarida is absent in Japygidae, but similar spheroidal ‘Trauben’ are present in all these taxa.

#### Central complex

The central complex of pterygote insects consists of a central body with a lower and upper division (also termed ellipsoid body and fan-shaped body, respectively), a protocerebral bridge, noduli and lateral accessory lobes [75]. Shape and connectivity of the central body in Diplura, as far as known, is reminiscent of the fan-shaped body of pterygotes. Neither an ellipsoid body, nor noduli could be identified. Regarding the lateral accessory lobes we explicitly do not rule out that alp and lal, as a whole, are the homologues in the dipluran brain (it is not clear whether Hanström’s ‘Nebenlappen’ (Table 1) correspond to lal, alp or both). The lateral neuropils alp and lal in Diplura are two distinct lobes, yet closely aligned and connected with each other. Thus they could be called lateral complex, a term used e.g. in flies [76]. The lobes directly connected to the central body are termed lal in this account. The immunoreactivity pattern of the central body, a TK-like ir layer sandwiched between two AS-like ir layers, is identical to the pattern in the fan shaped body of *Periplaneta americana*[4].

It has been shown in the locust *Schistocerca gregaria* that the eight columns of the upper central body division and the chiasmata of the eight w-, x-, y-, and z-tracts are generated by ‘fascicle switching’ between two commissures during embryonic development [77]. The w-, x-, y-, and z-tracts are formed by the progeny of eight neural stem cells by a special mode of amplifying neurogenesis that could be plesiomorphic for hexapods and crustaceans alike [78]. The division into 9 columns may seem unusual at first since generally the 8- or 16-fold organization of the protocerebral bridge and fan shaped body of pterygote insects is emphasized. However, the 16 fold organization of the locust protocerebral bridge (with eight w-, x-, y-, and z-tracts) can give rise to nine fold patterns in the central body: The CL1 (=CCI) neurons form a set of nine bundles in the upper division starting from 16 neurite bundles [55,79]. The outermost bundles run along the ventral surface of the central body, while the others cross over in groups of two, forming the main part of the seven posterior vertical bundles [55]. Furthermore, Golgi impregnation demonstrated (Figure two e of [27]) that 9 columns are formed by efferents in the fan-shaped body of the phasmatodean *Extatosoma tiaratum*. An unpaired median column, or at least a considerable amount of neuropil in the interstices between columns, is also present in the praying mantis *Tenodera aridifolia sinensis*[80] (p. 544) and in *Drosophila*[80] (p. 406). These examples suggest that the nine central body columns of Diplura could be formed by the same underlying plesiomorphic 8/16 fold organization by a different way of ‘packing’ them into columns. This mechanism, however, cannot explain the way the unpaired median column protrudes dorsally above the central body of Diplura. The protrusion of the unpaired median column of the central body is assessed an apomorphic character state in Diplura since it is not described in any other hexapod or crustacean group.

A comparison with other primarily wingless hexapods regarding the central complex shows high variation and the distribution of states that does not clearly reflect phylogenetic signal. Additionally, studies are in conflict about the presence or absence of components of the central complex. The central body of *Acerentomon maius* (Protura) does not show a distinct segmentation into eight columns as claimed by [81] for another *Acerentomon* species (AB, unpublished observation). Apart of the absence of distinct compartmentalization, the data presently available for the central complex of Protura remains very scarce. Collembola are reported to have a central body with eight columns (not as clearly separated as in Diplura) and a protocerebral bridge, while lacking noduli and a lower division of the central body [82]. However, Kollmann et al. [1], using immunostaining, found indications of noduli (in one collembolan) and of a lower central body division in two out of three investigated collembolan representatives.

Archaeognatha possess a homogeneous spindle-shaped central body that resembles the central body of certain decapod crustaceans [27,29]. According to [2] they have a protocerebral bridge connected to the central body by tracts which form a chiasma.

Loesel et al. [4] found three layers in the central body of the zygentoman *Lepisma saccharina*, the two lower ones of which were interpreted as ellipsoid and fan-shaped body, but no noduli. According to [32] the central body of *Lepisma saccharina* has a lower and an upper division without distinct columns. Likewise [75] refers to a lower and upper division for all Dicondylia, and an absence of noduli for non-pterygotes.

### Brain evolution in hexapods

Character polarization and reconstruction of the early evolution of characters of the hexapod brain often remains ambiguous. Since no studies have questioned the sister group relationship of Archaeognatha to Dicondylia, the greater resemblance of the central bodies of Diplura and Collembola [1] to pterygote fan-shaped bodies than to the archaeognathan central body, that is similar to the central body in certain decapod crustaceans [27], remains difficult to interpret. The total absence of mushroom bodies in Archaeognatha is even harder to explain. Essentially one of the following three scenarios, or combinations thereof, could explain these conflicts:

#### Independent acquisition of characters

Multiple acquisition of all brain characters discussed above for Collembola, Diplura and Dicondylia (Zygentoma + Pterygota) would at first glance appear quite unlikely. However, an 8-fold neuronal organization of the central body, without compartmentalization into columns, may be part of the ground pattern of hexapods, as is likewise assumed for decapod crustaceans [83]. The central body column formation in Diplura and Pterygota then may be the result of a convergent progression of a preexisting compartmentalization in these taxa.

Interestingly, a recent study suggests that the gene expression pattern for mushroom body formation possesses homologues in developmental gene cascades in the vertebrate pallium [84]. In this context it is conceivable that a plesiomorphic developmental program for mushroom body formation was switched on independently in Diplura, eventually in Collembola, and in Zygentoma + Pterygota. In this case, however, repression of the pathway in Archaeognatha is more parsimonious. The traditional rule of Dollo [85] states that complex structures, once lost, cannot be regained. Several studies give examples for possible violations of this rule. Ancestral polymorphism or other causes might be a better explanation in some cases, for example in the evolution of stick insect wings [86] (for critics see also [87]). Nevertheless, in a limited number of cases re-evolution of complex structures seems the best explanation for perceived character distribution [88].

#### Multiple character loss or reversal

Especially regarding mushroom bodies, secondary loss in Archaeognatha seems unlikely: On the one hand, mushroom body lobes are retained even in all examined anosmic pterygote insects [26]. On the other hand, there are several examples for adaptive loss in the central nervous system of arthropods [30,80]. Although loss of unnecessary neuronal processing capacity is highly favored energetically [89,90], in the case of Archaeognatha the behavioral repertoire and sensory organs seem as developed as in Zygentoma, for example. Thus it is not obvious why the energetic benefit of loosing mushroom bodies should outweigh the costs of presumably decreased functional capacity in Archaeognatha. The high similarity of Archaeognatha to decapod crustaceans in several observed traits might also be an indication for violations of the assumption of character independence. Such an interpretation awaits further studies especially on coupling mechanisms during the development of the central nervous system.

#### Effect of unsettled phylogenetic questions

One prerequisite for the discussion on organ evolution is the availability of a robust phylogeny for the examined species. This may not hold true for Diplura. Since the main problems in character polarization are due to observed states in the brain anatomy of Archaeognatha, errors in phylogenetic hypotheses seem to be less important in the present case: The sister group relationship of Archaeognatha and Dicondylia is well supported from both morphological and molecular studies. The open question on the relationship among entognathous hexapods has less implications for the polarization of currently known brain characters.

The relationships of crustacean subgroups and the position of Hexapoda within Crustacea are not yet unambiguously resolved, see e.g. [8,11,67,91]. Reliability of character polarizations will increase once more morphological and molecular data for all relevant subgroups of hexapods and crustaceans becomes available.

## Conclusions

Overall we confirm the findings of Hanström [32] and Holmgren [31] regarding the central complex, the mushroom bodies and the deutocerebrum of Diplura. A major difference is the discovery of two new mushroom bodies, mb2 in *Campodeaaugens* and mb5 in the investigated Japygidae, which were either not described or misinterpreted by Hanström and Holmgren. Among hexapods, the mb5 in Diplura is the only known mushroom body that extends across the midline. The central body with a three layered organization appears reminiscent to that in *Periplaneta americana*, although a protruding median column is present in all investigated diplurans. In future studies the inclusion of more taxa, especially Projapygoidea, the use of methods appropriate to visualize single neurons and further antibody staining may provide additional insights into brain evolution at the base of the phylogenetic tree of hexapods.

## Competing interests

The authors declare that they have no competing interests.

## Authors’ contributions

AB did the experimental work and drafted the manuscript. GP initiated the study and like NUS contributed to writing. All authors read and approved the final manuscript.
